# The Modification of H3K4me3 Enhanced the Expression of *Cg*TLR3 in Hemocytes to Increase *Cg*IL17-1 Production in the Immune Priming of *Crassostrea gigas*

**DOI:** 10.3390/ijms25021036

**Published:** 2024-01-15

**Authors:** Xingye Lian, Yinan Li, Weilin Wang, Jiajun Zuo, Tianqi Yu, Lingling Wang, Linsheng Song

**Affiliations:** 1 School of Life Science, Liaoning Normal University, Dalian 116029, China; lianxingye@foxmail.com (X.L.); liyinan0703@163.com (Y.L.); 2 Liaoning Key Laboratory of Marine Animal Immunology and Disease Control, Dalian Ocean University, Dalian 116023, China; wangweilin@dlou.edu.cn (W.W.); z948167604@163.com (J.Z.); yutianqiswgc@163.com (T.Y.); 3 Southern Marine Science and Engineering Guangdong Laboratory (Zhuhai), Zhuhai 519000, China; 4 Laboratory of Marine Fisheries Science and Food Production Process, Qingdao National Laboratory for Marine Science and Technology, Qingdao 266235, China; 5 Dalian Key Laboratory of Aquatic Animal Disease Prevention and Control, Dalian Ocean University, Dalian 116023, China

**Keywords:** H3K4me3, immune priming, interleukin 17, Toll-like receptor, *Crassostrea gigas*

## Abstract

Increasing evidence confirms that histone modification plays a critical role in preserving long-term immunological memory. Immune priming is a novel form of immunological memory recently verified in invertebrates. Toll-like receptor (TLR) signaling and cytokines have been reported to be involved in the immune priming of the Pacific oyster *Crassostrea gigas*. In the present study, the expression of Toll-like receptor 3 (*Cg*TLR3), myeloid differentiation factor 88-2 (*Cg*Myd88-2) and interleukin 17-1 (*Cg*IL17-1) was found to be elevated in the hemocytes of *C. gigas* at 6 h after the secondary stimulation with *Vibrio splendidus*, which was significantly higher than that at 6 h after the primary stimulation (*p* < 0.05). A significant increase in histone H3 lysine 4 trimethylation (H3K4me3) enrichment was detected in the promoter region of the *Cg*TLR3 gene at 7 d after the primary stimulation with inactivated *V. splendidus* (*p* < 0.05). After the treatment with a histone methyltransferase inhibitor (5′-methylthioadenosine, MTA), the level of H3K4me3 at the promoter of the *Cg*TLR3 gene decreased significantly at 7 d after the primary stimulation with inactivated *V. splendidus* (*p* < 0.05), and the expression of *Cg*TLR3, *Cg*MyD88-2 and *Cg*IL17-1 was significantly repressed at 6 h after the secondary stimulation with *V. splendidus* (*p* < 0.05). Conversely, the treatment with monomethyl fumarate (MEF, an inhibitor of histone demethylases) resulted in a significant increase in H3K4me3 enrichment levels at the *Cg*TLR3 promoter at 7 d after the primary stimulation (*p* < 0.05), and the expression of *Cg*TLR3, *Cg*MyD88-2 and *Cg*IL17-1 was observed to increase significantly at 6 h after the secondary stimulation (*p* < 0.05). These results suggested that H3K4me3 regulated MyD88-dependent TLR signaling in the hemocytes of *C. gigas*, which defined the role of histone modifications in invertebrate immune priming.

## 1. Introduction

The immune systems of invertebrates can mount an immune memory response to subsequent reinfections with the same or different pathogens, which is defined as immune priming [1,2]. In contrast to the adaptive immunity in vertebrates that primarily depends on the rearrangement of immunoglobulin family genes and clonal expansion, the immune priming in invertebrates may be mainly governed by epigenetic modification [3,4,5,6]. Epigenetic programming usually occurs as a result of viral and bacterial infections, the stimulation of pathogen-associated molecular patterns (PAMPs), and the induction of various cytokines. Epigenetic modification plays a crucial role in tightly regulating pathogen recognition, intracellular signal transduction, and downstream effector activation, which serves as a form of cellular memory in immune cells. It bestows distinct functions and specialized phenotypes upon immune cells through establishing cell-specific gene expression patterns. These epigenetic reprogramming events contribute to the biological functions of primed cells and the enhancement of their immune responses [7,8].

Epigenetic modifications are heritable changes in chromatin structure and gene expression without alterations in the DNA sequence. Major epigenetic modifications include histone methylation, acetylation and phosphorylation, DNA methylation, and microRNA modifications. Trimethylation of histone H3 lysine 4 (H3K4me3) is a common and evolutionarily conserved epigenetic modification, which is known to be associated with transcriptionally active chromatin [9]. It is widely recognized that the transcriptional activity of a gene positively correlates with the degree of H3K4me3 at its promoters. The presence of H3K4me3 maintains the chromatin in a conformation that facilitates accessibility of the promoter region for specific genes, thereby promoting transcriptional activity [10]. In mammals, several investigations have highlighted the pivotal significance of the H3K4me3 modification in innate immunity as well as the “trained immunity” [11,12,13,14]. Trained immunity is a functional adaptation of mammalian innate immune cells induced by epigenetic reprogramming, resulting in an enhanced immunologic response [15]. The H3K4me3 modification has been implicated in various forms of trained immunity of immune cells, including lipopolysaccharide-trained dendritic cells, oxidized low-density lipoprotein-trained monocytes, catecholamines-trained monocytes, and interleukin-trained natural killer cells [16,17,18,19]. Systematic chromatin reprogramming has been reported in β-Glucan-trained human monocytes, which is characterized by elevated levels of H3K4me3 at the gene promoters [20]. The H3K4me3 modification is necessary for the enhanced release of cytokines in the trained monocytes after Bacillus Calmette–Guerin (BCG) vaccination in human [12]. It is believed that H3K4me3 modification is of utmost importance in preserving the delicate balance of the innate immune response by increasing the expression of specific pattern recognition receptors (PRRs) and some immune effectors. However, H3K4me3 modification and its role in the immune priming of invertebrate are still not well understood.

Immune recognition is the first step in the immune response to foreign invaders, which is initiated by binding and interaction between host PRRs and PAMPs from invasive pathogens. The Toll-like receptor (TLR) is a type of PRR that is widely expressed by immune cells to mediate immune signaling. Among the various pathways, TLR signaling has been extensively investigated and the mechanism of signal transduction is well documented. After binding with a PAMP, the TLR initiates signaling transduction by recruiting its canonical adaptor such as myeloid differentiation factor 88 (MyD88) to activate various transcription factors such as nuclear factor nuclear factor kappa-B (NF-κB), activating protein-1 and interferon regulatory factors, and eventually induce immune responses [21,22,23]. An increase in H3K4me3 levels was observed on the promoters of many TLR signaling molecules and cytokines such as TLR4, scavenger receptor A, interleukin (IL) 6 and tumor necrosis factor (TNF) α in trained immunity [17,24,25,26]. Histone methyltransferases, such as KMT2B and ASH1L, were reported to modulate the TLR4-mediated signaling in macrophages by increasing the H3K4me3 levels of TLR ligands or regulators [14,27]. For invertebrates, more research is needed to clarify the histone modifications and other epigenetic modifications on PRRs and downstream signaling pathways during immune responses.

Cytokines are a diverse group of soluble proteins that regulate the development, differentiation, and activation of immune cells to modulate both innate and adaptive immune responses. Several proinflammatory cytokines, such as IL1, IL6, IL1β, and TNFα have been extensively investigated in the context of trained immunity [12,20,28]. To date, most vertebrate proinflammatory cytokines, including IL1, IL2, and IL6, as well as their corresponding receptors, have not been identified in invertebrates [29]. Recently, IL17 signaling components were annotated and identified in bivalves [30], and they were reported to play important roles in the initiation of the proinflammatory response and other immune responses in the Pacific oyster *Crassostrea gigas* [31,32,33,34,35]. Considering that proinflammatory cytokines have been extensively investigated in the trained immunity of vertebrate, IL17 signaling components are also suspected to play a crucial role in oyster immune priming.

The Pacific oyster *C. gigas* is a worldwide aquaculture animal, which belongs to the second largest animal phylum Mollusca. As a sessile filter-feeder exposed to numerous microorganisms, the oyster represents an attractive model for studying immune mechanisms in invertebrates. The oyster mainly relies on the innate immune system to defend against infection by pathogens [36,37]. Increasing evidence demonstrates the presence of immune priming in *C. gigas*, and many key components of PRR signaling pathways are suspected to be involved in immune priming [35,38,39,40,41]. At the same time, H3K4me3 has also reported to be crucially involved in regulating the signaling pathways in trained immunity [7,12,20,25]. The TLR-MyD88-NF-κB signaling has been reported to govern the expression of inflammatory cytokines [32,42,43,44] and play an indispensable role in the enhanced immunity against re-infection of *Vibrio splendidus* [35]. TLR3 from *C. gigas* was upregulated after both the first and second immune stimulations, indicating that it was involved in immune priming [35]. It was reported that *Cg*TLR3 was able to interact with *Cg*MyD88-2 [45], and *Cg*Rel1 functioned as an important transcription factor, regulating the expression of *Cg*IL17s in the immune responses of oysters [46]. In the present study, the effect of H3K4me3 on TLR expression in oysters was investigated with the objectives of (1) examining the responses of *Cg*IL17-1 and TLR signaling in hemocytes after the secondary immune stimulation with *V. splendidus*, (2) measuring the H3K4me3 modification at the *Cg*TLR3 promoter after the stimulations with of *V. splendidus*, 5′-methylthioadenosine (MTA, a methyltransferase inhibitor) and Monoethyl fumarate (MEF, a demethylase inhibitor), respectively, and (3) determining the changes in TLR signaling and *Cg*IL17-1 mRNA expression after the treatment with MTA.

## 2. Results

### 2.1. The mRNA Expression Levels of CgTLR3, CgMyD88-2, CgRel1, and CgIL17-1 at 6 h after the Secondary Stimulation with Live V. splendidus

**Figure 1:**
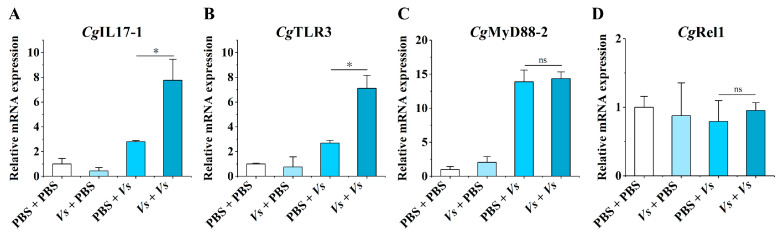
The mRNA expression levels of *Cg*TLR3, *Cg*MyD88-2, *Cg*Rel1, and *Cg*IL17-1 at 6 h after the secondary stimulation with live *V. splendidus* (*Vs*). The relative mRNA expression levels of (**A**) *Cg*IL17-1, (**B**) *Cg*TLR3, (**C**) *Cg*MyD88-2, and (**D**) *Cg*Rel1 in the four groups (PBS + PBS, *Vs* + PBS, PBS + *Vs*, *Vs* + *Vs*) were determined by qPCR. Vertical bars represent the mean ± SD (*N* = 3). *: *p* < 0.05, ns: no significant difference (*t*-test).

Based on the transcriptome analysis and annotation of TLRs and ILs in oyster hemocytes after the secondary immune stimulation with live *V. splendidus*, the mRNA transcripts of *Cg*TLR3, *Cg*MyD88-2, *Cg*Rel1, and *Cg*IL17-1 were selected and examined in the four treatment groups (PBS + PBS, *Vs* + PBS, PBS + *Vs*, *Vs* + *Vs*) by RT-qPCR (Figure 1). The mRNA transcripts of *Cg*IL17-1 were detected in all four groups with the highest expression level in the *Vs* + *Vs* group, which was 2.77-fold (*p* < 0.05) of that in the PBS + *Vs* group (Figure 1A). The *Cg*TLR3 mRNA expression level in the *Vs + Vs* group was significantly increased (2.64-fold, *p* < 0.05) compared to that in the PBS + *Vs* group (Figure 1B). The relative mRNA expression level of the *Cg*MyD88-2 in *Vs* + *Vs* group was 1.03-fold of that in PBS + *Vs* group, with no significant differences (Figure 1C). There was no significant difference in the *Cg*Rel1 mRNA expression levels among the four treatment groups (Figure 1D).

### 2.2. The H3K4me3 Modification of the CgTLR3 Gene Promoter at 7 d after the Stimulation with Inactivated V. splendidus

**Figure 2:**
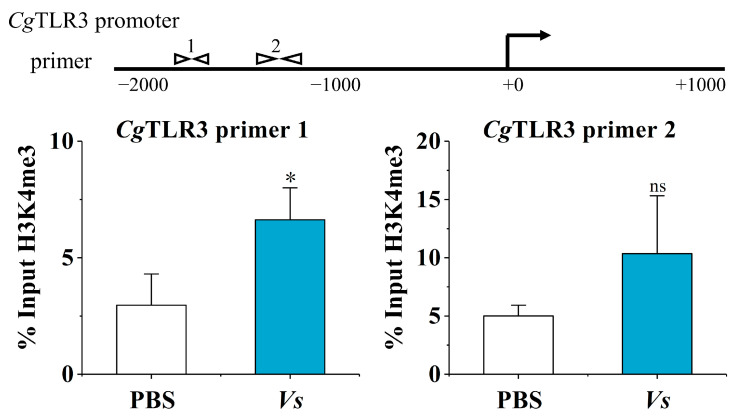
The H3K4me3 modification levels of the *Cg*TLR3 gene promoter at 7 d after the stimulation with inactivated *V. splendidus* (*Vs*). The H3K4me3 modification levels of the *Cg*TLR3 gene promoter were determined by ChIP-qPCR. The positions of the primers used to examine H3K4me3 modification at the promoter regions of *Cg*TLR3 are shown in the top panel of the figure. Vertical bars represent the mean ± SD (*N* = 3). *: *p* < 0.05, ns: no significant difference (*t*-test).

The H3K4me3 modification levels of the *Cg*TLR3 promoter at 7 d after the stimulation with inactivated *V. splendidus* were examined using chromatin immunoprecipitation followed by qPCR (ChIP-qPCR). The primers were designed using Primer Premier 5 to cover the *Cg*TLR3 promoter region (Figure 2). ‘% input H3K4me3’ indicates the ratio of the DNA fragments of each promoter region bound by H3K4me3 to the total amount of input DNA fragments without H3K4me3 antibody pull-down. At 7 d after the stimulation with inactivated *V. splendidus*, anti-H3K4me3 antibodies markedly enriched *Cg*TLR3 promoter DNA. Compared to the PBS group, the H3K4me3 modification level of *Cg*TLR3 distal promoter regions (spanning from −1879 bp to −1735 bp) was significantly higher in the *Vs* group (2.24-fold, *p* < 0.05), and the proximal promoter regions −1307 bp to −1164 of *Cg*TLR3 also showed a slightly higher H3K4me3 modification level in the *Vs* group, but this difference did not reach significance (2.07-fold, *p* = 0.14).

### 2.3. The Alternation of H3K4me3 Modification Levels at the CgTLR3 Gene Promoter at 7 d after the Treatments with MTA and MEF

**Figure 3:**
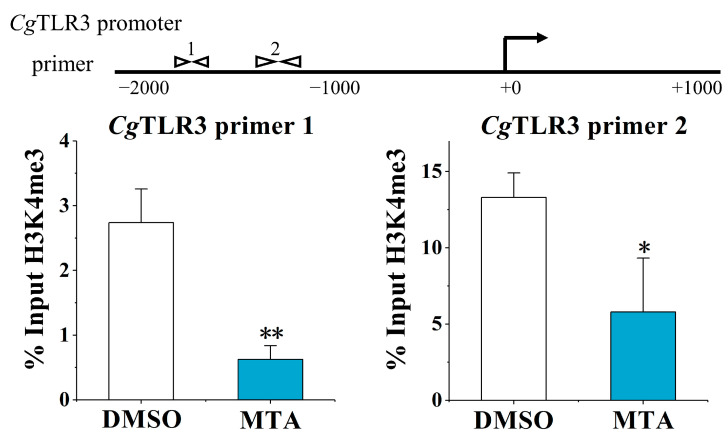
The H3K4me3 modification levels of the *Cg*TLR3 gene promoter at 7 d after the histone methyltransferase inhibitor (MTA) treatment. The H3K4me3 modification levels of the *Cg*TLR3 gene promoter were determined by ChIP-qPCR. The positions of the primers used to examine H3K4me3 modification at the promoter regions of *Cg*TLR3 are shown in the top panel of the figure. Vertical bars represent the mean ± SD (*N* = 3). *: *p* < 0.05, **: *p* < 0.01 (*t*-test).

**Figure 4:**
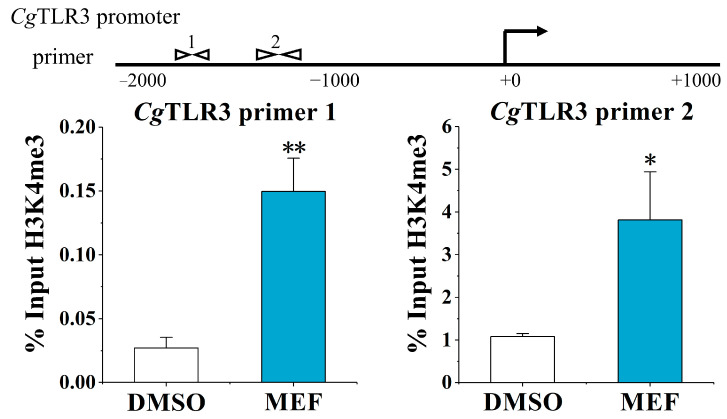
The H3K4me3 modification levels of the *Cg*TLR3 gene promoter at 7 d after the histone demethylase inhibitor (MEF) treatment. The H3K4me3 modification levels of the *Cg*TLR3 gene promoter were determined by ChIP-qPCR. The positions of the primers used to examine H3K4me3 modification at the promoter regions of *Cg*TLR3 are shown in the top panel of the figure. Vertical bars represent the mean ± SD (*N* = 3). *: *p* < 0.05, **: *p* < 0.01 (*t*-test).

The H3K4me3 modification levels of the *Cg*TLR3 promoter were examined at 7 d after the stimulation with the methylation inhibitor MTA and histone demethylases inhibitor MEF. Upon the MTA treatment, the H3K4me3 modification levels at the *Cg*TLR3 gene promoter were significantly lower: between primer pair −1879 and −1735 (0.23-fold, *p* < 0.01, primer 1) and primer pair −1307 and −1164 (0.44-fold, *p* < 0.05, primer 2), compared with the DMSO group, respectively (Figure 3). The MEF treatment also resulted in a 6.68-fold enrichment of *Cg*TLR3 distal promoter DNA using primer 1 (*p* < 0.01) and a 3.54-fold enrichment of *Cg*TLR3 proximal promoter DNA using primer 2 (*p* < 0.05), compared with the DMSO group, respectively (Figure 4).

### 2.4. The mRNA Expression of CgTLR3, CgMyD88-2, CgRel1, and CgIL17-1 at 6 h after the Secondary Stimulation upon the MTA and MEF Treatment

**Figure 5:**
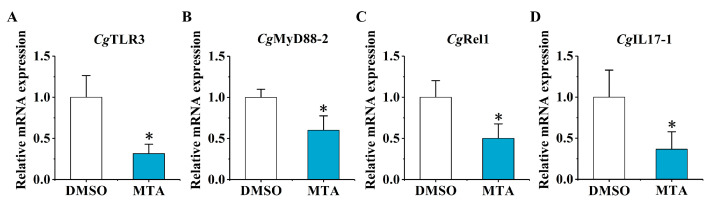
The mRNA expression levels of *Cg*TLR3, *Cg*MyD88-2, *Cg*Rel1, and *Cg*IL17-1 at 6 h after the secondary stimulation upon the MTA treatment. The relative mRNA expression levels of (**A**) *Cg*IL17-1, (**B**) *Cg*TLR3, (**C**) *Cg*MyD88-2, and (**D**) *Cg*Rel1 in the MTA group and the DMSO group were determined by qPCR. Vertical bars represent the mean ± SD (*N* = 3). *: *p* < 0.05 (*t*-test).

**Figure 6:**
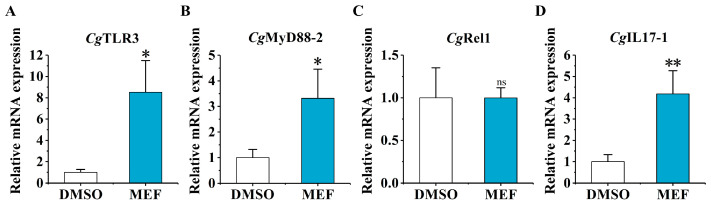
The mRNA expression levels of *Cg*TLR3, *Cg*MyD88-2, *Cg*Rel1, and *Cg*IL17-1 at 6 h after the secondary stimulation upon the MEF treatment. The relative mRNA expression levels of (**A**) *Cg*IL17-1, (**B**) *Cg*TLR3, (**C**) *Cg*MyD88-2, and (**D**) *Cg*Rel1 in the MEF group and the DMSO group were determined by qPCR. Vertical bars represent the mean ± SD (*N* = 3). *: *p* < 0.05, **: *p* < 0.01, ns: no significant difference (*t*-test).

The relative mRNA expression levels of *Cg*TLR3, *Cg*MyD88-2, *Cg*Rel1, and *Cg*IL17-1 were examined at 6 h after the secondary stimulation in the MTA group and the MEF group by RT-qPCR. Upon the MTA treatment, the mRNA expression levels of *Cg*TLR3, *Cg*MyD88-2, *Cg*Rel1, and *Cg*IL17-1 were significantly repressed by 31.45% (*p* < 0.05), 60.06% (*p* < 0.05), 50.06% (*p* < 0.05), and 36.60% (*p* < 0.05), compared with the DMSO group, respectively (Figure 5). In the MEF group, the relative mRNA expression levels of *Cg*TLR3, *Cg*MyD88-2, and *Cg*IL17-1 increased significantly (8.52-fold, *p* < 0.05, 3.32-fold, *p* < 0.05, and 4.18-fold, *p* < 0.01, compared with the DMSO group, respectively), while the mRNA transcripts of *Cg*Rel1 showed no significant change (Figure 6).

## 3. Discussion

Epigenetic modifications play an important role in the maintenance of immunological memory. To date, knowledge of the epigenetic regulation mechanism in invertebrate immune priming has remained very limited. The circulatory hemocytes in invertebrates are considered counterparts of vertebrate monocytes, which are responsible for the immune response, as well as immune priming [36,47]. In our previous study, enhanced phagocytosis and the promoted regeneration of circulating hemocytes were found in primed oysters when they encountered the secondary challenge with *V. splendidus*, which indicated that hemocytes play important roles in oyster immune priming [41]. Oyster hemocytes are important immune effector cells that are thought to participate in phagocytosis and the secretion of cytokines [36]. In the present study, the prestimulation of *C. gigas* with *V. splendidus* induces an H3K4me3 modification of the *Cg*TLR3 promoter that modulates the mRNA expression levels of *Cg*TLR3, *Cg*MyD88-2, *Cg*Rel1, and *Cg*IL17-1 in hemocytes after the secondary immune stimulation with *V. splendidus*. The epigenetic treatment with MTA and MEF could affect the H3K4me3 modification of the *Cg*TLR3 promoter as well as the *Cg*TLR3 and *Cg*IL17-1 mRNA expression in oyster hemocytes.

A number of pro-inflammatory cytokines have been proposed to play critical roles in trained immunity. The increased production of IL6, IL18, IL1β, or TNFs was used as key indicator for the detection of trained immunity in vertebrates [12,48,49]. Some cytokines such as IL17s [31], TNFs [50], and interferons have been identified in *C. gigas* [29,51,52]. There are ten IL17 genes annotated from the genome of *C. gigas*, which are considered to play diverse roles in immune defense [30]. Several reports have shown that *Cg*IL17-1 is involved in inflammatory mobilization and hemocyte proliferation in the innate immune response of *C. gigas* [33,34,53]. In the present study, in order to find the cytokines with enhanced expression in immune priming, we analyzed the previously reported transcriptome data [35] and identified *Cg*IL17-1 as a key cytokine for further investigation. Increased transcripts of *Cg*IL17-1 upon restimulation were observed (Figure 1A), which confirms the role of *Cg*IL17-1 in oyster immune priming. These results are in agreement with the available evidence that the IL family is associated with immunological memory. For instance, a review article suggested that IL1 family members, especially IL1β, are crucial components of trained immunity [28]. Studies in mice demonstrated that the training of monocytes led to enhanced production of pro-inflammatory cytokines TNF-α and IL6 [20]. In teleost fish, *Scophthalmus maximus*, trained neutrophils exhibited a significant elevation of the IL1R signaling pathway after *Edwardsiella piscicida* infection [54]. Hence, it could conceivably be hypothesized that *Cg*IL17-1 is an important component of oyster immune priming, which warrants further study.

The recognition of PAMPs through TLRs induces a series of signaling events that result in an acute inflammatory response. The binding of the TLR to ligands is known to trigger the MyD88-dependent NF-κB pathway, which activates the expression of proinflammatory genes such as TNFs and ILs in the innate response of vertebrates [55,56]. In mollusks, TLRs also shown to activate MyD88 and induce IL17 expression [57,58,59]. Studies have reported the existence of the TLR-MyD88-NF-κB signaling axis, which regulates the expression of inflammatory cytokines such as IL17s and TNFs in oysters [32,42,43,44]. Of note, compared to the finding that mammals typically possess 13 TLRs, 83 TLRs were identified in the genome of *C. gigas* and shown to be strain-specific, possibly because of oyster-specific immune adaptations [41]. Recently, *Cg*TLR3 was reported to be involved in oyster immune defense by a MyD88-dependent NF-κB pathway [45], and to regulate *Cg*IL17-1 expression [60]. In the present study, *Cg*TLR3 was identified as a key PRR for further investigating its role in immune priming. The enhanced expression of *Cg*TLR3 in the second immune response of oysters was validated, which could further promote the enhanced expression of *Cg*IL17-1. Studies have demonstrated that TLRs play a crucial role in the initiation of immunological memory [23,61]. Various microbial components induce the activation of dendritic cells through TLR signals, which leads to T-cell priming and an acquired immune response [62,63,64]. In *Drosophila melanogaster*, the Toll pathway is required for enhanced protection against *Streptococcus pneumonia* re-infection [65]. In crab *Eriocheir sinensis*, the *Es*TLR1-mediated productions of *Es*ALF-1 and *Es*ALF-3 in hemolymph played an indispensable role in the month-long humoral immune protection induced by *Aeromonas hydrophila*. In mud crab *Scylla paramamosain*, the TLR pathway played an important role in enhanced immune protection against reinfection by *Vibrio parahaemolyticus* [66]. In Pacific abalone *Haliotis discus hannai*, the IL17 and TLR signaling pathway were likely dominant in the immune enhancement process in response to the re-infection of *V. parahaemolyticus* [67]. Our previous transcriptomic data also suggested higher expression levels of *Cg*MyD88 and *Cg*TIMP after the second stimulation with *V. splendidus*, indicating the key roles of *Cg*TLR3 signaling in oyster immune priming [35]. According to these data, we can infer that *Cg*TLR3 was involved in immune priming and contributes to inducing MyD88-dependent NF-κB pathway activation and IL17-1 expression in oyster hemocytes, implying the indispensable role of the TLR signaling pathway in enhanced immune protection. Though it is conserved for TLR3 in activating the MyD88-dependent NF-κB pathway, the exact roles of this pathway in the immune priming of oysters needs further investigation.

The epigenetic modifications that facilitate gene transcription have been demonstrated as an important mechanism that influences the trained immune response to secondary stimulation in vertebrates [8]. For invertebrates, the possible epigenetic mechanisms of gene expression related to innate immune memory were recently proposed [68,69], in which histone modifications have been recognized as pivotal players. It was found that *Anopheles gambiae* and *Aedes aegypti* possess an innate immune memory maintained by histone acetyltransferase [70,71]. H3K4me3 is a histone modification exclusively found at active promoters and is therefore often enriched in promoter regions regulating TLRs [72]. It was found that TLR4 expression is regulated by H3K4me3 on its promoter in diabetic macrophages and wound myeloid cells [24,73]. In *C. gigas*, H3K4 methylation modifications have been reported to be crucial for the hematopoiesis and the embryonic development process [74,75,76]. In this study, the inactivated bacteria enriched H3K4me3 modification at the *Cg*TLR3 promoter regions. Moreover, the epigenetic drugs MTA and MEF can change the H3K4me3 enrichment of the *Cg*TLR3 promoter, and further affect the mRNA expression of *Cg*MyD88-2 and *Cg*IL17-1. These results suggested that H3K4me3 was involved in the regulation of TLR3 expression and IL17-1 production in hemocyte-mediated immune priming in oysters. It was found that the oysters primed with the stimulation of *V. splendidus* exhibited enhanced phagocytosis and the regeneration of circulating hemocytes when they encountered the second stimulation with *V. splendidus* [41]. Studies have suggested that different PRRs and associated molecules were over-represented after the secondary challenge. Several PRRs, such as Down syndrome cell adhesion molecules in crustaceans and insects [77], the variable lymphocyte receptor in crab *E. sinensis* [78], fibrinogen-related proteins in vector snail *Biomphalaria glabrata* [79], C-lectins in scallop *Chlamys farreri* [80], have been implicated in the innate immune memory of invertebrates. Speculative priming in the invertebrate immune system may be caused by synergistic interactions and dosage effects within the immune system, which is more complicated than expected [81]. It is generally assumed that the duration of immune priming can range from 7 to 30 d in different species with different pathogens [82]. The antiviral immune priming phenomenon in oysters was reported to be long-lasting, persisting for at least 5 months [83]. Besides TLRs and H3K4me3, there is abundant room for further progress in determining the detailed mechanism in the immune priming of oysters.

In conclusion, the regulatory mechanism of H3K4me3 in the immune priming of oyster hemocytes was investigated. The TLR expression can be regulated by H3K4me3 methylation in its promoter, which may further affect the enhanced MyD88-dependent TLR signaling transduction and IL expression against the second stimulation with *V. splendidus*. All the results suggested that H3K4me3 was involved in the immune priming of *C. gigas* by regulating the mRNA expression of TLR signaling molecules, which offer valuable insights for the regulatory mechanisms of immune memory in invertebrates.

## 4. Materials and Methods

### 4.1. Experimental Animals and Bacteria

The oysters *C. gigas* used in the present study were about two years old, and their shell length was of 12–16 cm. All the oysters were collected from an aquaculture farm in Dalian, Liaoning Province, China, and temporarily cultivated in filtered seawater at ambient temperature. The seawater was aerated using an air pump during the laboratory cultivation. The oysters were fed with concentrated algal powder daily and the water was completely replaced once a day. All oyster experiments were performed in accordance with the approval and guidelines of the Ethics Review Committee of Dalian Ocean University.

*V. splendidus*, isolated from lesion-like niduses of the moribund scallop *Patinopecten yessoensis*, was employed to stimulate oysters as previously described [84]. It was cultured in 2116E media at 18 °C for 24 h, harvested by centrifugation at 4000× *g* for 10 min, resuspended in phosphate-buffered saline (PBS), and adjusted to the final concentration of 2 × 10^8^ CFU mL^−1^.

### 4.2. Immune Priming Induction and Hemocyte Collection

**Figure 7:**
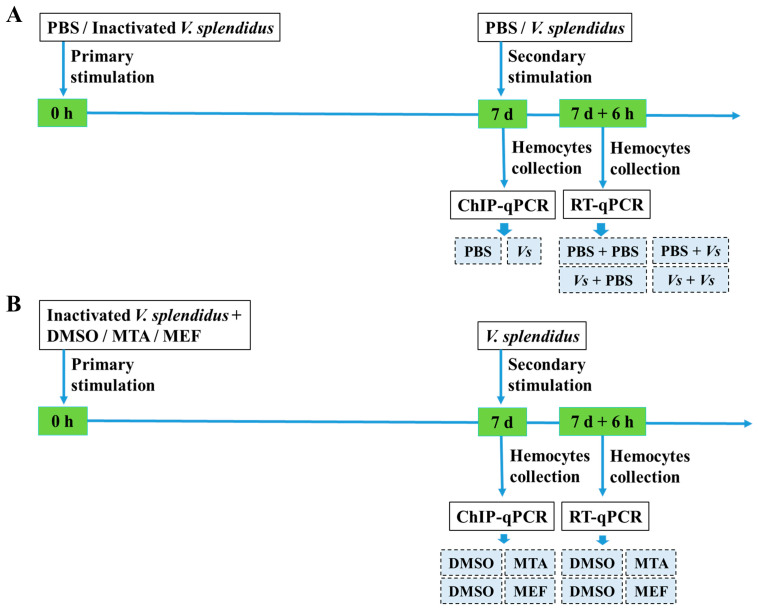
Schematic representation of the experimental design used to measure H3K4me3 modification levels of genes promoter and mRNA expression levels in oyster hemocytes. (**A**) Detailed information about the experimental design pertaining to primary and secondary stimulation for immune priming study. The same batch of oysters was injected with inactivated *V. splendidus* (*Vs*) or PBS, and at 7 d after the first immune stimulation, the oysters were restimulated with *Vs* or PBS for 6 h. Hemolymph samples were collected at 7 d after the first immune stimulation and at 6 h after the secondary stimulation for the following experiment. (**B**) Experimental design for epigenetic treatment. MTA, MEF, or DMSO was injected together with inactivated *V. splendidus*, and at 7 d after the first stimulation, the oysters were restimulated with live *V. splendidus* for 6 h. Hemolymph samples were collected at 7 d after the first immune stimulation and at 6 h after the secondary stimulation for the following experiment.

The bacterial stimulation experiment was conducted as previously described [85]. Fifty-four oysters were employed and divided equally into six groups designated as the PBS, *Vs*, PBS + PBS, PBS + *Vs*, *Vs* + PBS, and *Vs* + *Vs* groups (Figure 7A). In the PBS and *Vs* groups, the oysters individually received an injection of 100 μL of PBS or 100 μL of heat-killed *V. splendidus* (2 × 10^8^ CFU mL^−1^). At 7 d after the bacterial injection, nine oysters were randomly selected from each group and hemolymph samples were collected to examine the H3K4me3 modification levels of the *Cg*TLR3 gene promoter. In the PBS + PBS and PBS + *Vs* groups, the oysters received a first injection with 100 μL of PBS and a second injection with 100 μL of PBS or 100 μL of live *V. splendidus* (2 × 10^8^ CFU mL^−1^) at 7 d after the first injection, respectively. In the *Vs* + PBS and *Vs* + *Vs* groups, the oysters were first stimulated with 100 μL of heat-killed *V. splendidus*, and then treated with 100 μL of PBS and 100 μL of live *V. splendidus* as the second stimulation at 7 d after the first injection, respectively. The hemolymph samples from the PBS + PBS, PBS + *Vs*, *Vs* + PBS, and *Vs* + *Vs* groups were collected at 6 h after the second immune stimulation to examine the mRNA transcripts of *Cg*IL17-1, *Cg*TLR3, *Cg*MyD88-2, and *Cg*Rel1 (Figure 7A).

The hemolymph (about 0.5 mL from each oyster) was aseptically withdrawn from the posterior adductor muscle sinus, and the hemolymph collected from three oysters was mixed together as one sample. There were three replicates for each group. The hemocytes were harvested by centrifugation immediately at 800× *g*, 4 °C for 10 min.

### 4.3. The Treatments of MTA and MEF

Seventy-two oysters were employed for the treatment with MTA (the nonselective methyltransferase inhibitor, Sigma) and MEF (the histone demethylases inhibitor, Sigma). The oysters in the MTA group (18 oysters) and the MEF group (18 oysters) received injection of MTA and MEF at a dose of 96 μmol kg^−1^ body weight [86] and 50 mg kg^−1^ body weight [13], respectively. Thirty-six oysters treated with 100 μL of dimethyl sulfoxide (DMSO, 5% in PBS) were used as a control. After the treatments with MTA and MEF, 100 μL of heat-killed *V. splendidus* was injected immediately into each oyster. Hemolymph samples were collected from 36 oysters to examine the H3K4me3 modification level of the *Cg*TLR3 gene promoter at 7 d after the epigenetic treatment. At the same time, the secondary stimulation with 100 μL of live *V. splendidus* was conducted with the remaining 36 oysters. The mRNA transcripts of *Cg*IL17-1, *Cg*TLR3, *Cg*MyD88-2, and *Cg*Rel1 were examined at 6 h after the secondary stimulation.

### 4.4. RNA Isolation and cDNA Synthesis

Total RNA from oyster hemocytes was extracted using Trizol reagent (Invitrogen, Carlsbad, CA, USA) according to the manufacturer’s instructions. Nanodrop 2000 (Thermo Fisher Scientific, Waltham, MA, USA) and the Agilent 2100 Bioanalyzer (Agilent Technologies, Santa Clara, CA, USA) were used to determine the quantity and purity of the extracted RNA. The cDNA synthesis was performed using PrimeScript™ RT reagent Kit with gDNA Eraser (Takara, Dalian, China) according to the manufacturer’s instruction, and the cDNA mix was diluted 1:40 and stored at −80 °C for the subsequent fluorescent real-time quantitative PCR.

### 4.5. Reverse Transcription-Quantitative PCR (RT-qPCR) Analysis

The mRNA transcripts of *Cg*IL17-1, *Cg*TLR3, *Cg*MyD88-2, and *Cg*Rel1 were measured using specific primers (Table 1) with the elongation factor of *C. gigas* (*Cg*EF, CGI_10012474) as an internal control. The promoter sequences of the target genes were obtained from the NCBI database (http://www.ncbi.nlm.nih.gov, accessed on 9 September 2021), and Primer premier 6.0 software was used to design the primers for ChIP-qPCR (Table 1).

The PCR reactions were performed with the SYBR premix ExTap (RR420, Takara, Dalian, China) using the ABI PRISM 7500 Sequence Detection System (Thermo Fisher Scientific, Waltham, MA, USA) according to the manual. The relative expression levels were analyzed by comparative Ct method (2^−ΔΔCt^ method) [87].

### 4.6. ChIP-qPCR Assays

Chromatin immunoprecipitation in oyster hemocytes was conducted as previously described [85]. ChIP-qPCR assay was performed using a ChIP Assay Kit (Beyotime, Shanghai, China) according to the manufacturer’s instructions. The collected hemocytes were resuspended in the modified Leibovitz L-15 medium (supplemented with 20.2 g L^−1^ NaCl, 0.54 g L^−1^ KCl, 0.6 g L^−1^ CaCl_2_, 1.0 g L^−1^ MgSO_4_, 3.9 g L^−1^ MgCl_2_, 20.8 g L^−1^ glucose, 10% FCS, 100 mg mL^−1^ penicillin G, 100 mg mL^−1^ streptomycin, 40 mg mL^−1^ gentamicin and 0.1 mg mL^−1^ amphotericin B, pH 7.0) and formaldehyde (1% final concentration) was added into the hemocyte suspension to crosslink the DNA and protein. Hemocytes were lysed in SDS lysis buffer, and the cross-linked DNA was sonicated for 10 min to obtain DNA fragments around 250 bp. The cross-linked fragmented DNA was precleared with protein A agarose/salmon sperm DNA and the precleared DNA was incubated with H3K4me3 antibodies (Beyotime, Shanghai, China) at 4 °C overnight. The immunoprecipitates were then incubated with protein A/G agarose/salmon sperm DNA, and the DNA-histone complexes with salmon sperm DNA/protein G agarose beads were collected. The resultant immune complexes were successively washed once with low-salt buffer (150 mM NaCl, 0.1% SDS, 1% Triton X-100, 2 mM EDTA, 20 mM TRIS), once with high-salt buffer (500 mM NaCl, 0.1% SDS, 1% Triton X-100, 2 mM EDTA, 20 mM TRIS), once with LiCl buffer (0.25 M LiCl, 1% NP-40, 1% Na-deoxycholate, 1 mM EDTA, 10 mM TRIS), and twice with TE buffer. The cross-linked fragmented DNA was eluted by using elution buffer (0.1 M NaHCO_3_, 1% SDS). The same amount of cross-linked fragmented DNA without antibody precipitation was processed in the same manner and served as an input control. The cross-linked DNA was de-crosslinked with 200 mM sodium chloride at 65 °C for 4 h and the proteins were removed by treatment with proteinase K. The resultant DNA was extracted using the phenol/chloroform/isoamyl alcohol method. The primer sets used for the ChIP-qPCR were listed in Table 1.

### 4.7. Statistical Analysis

All data were given as means ± standard deviation (*N* = 3) and processed using SPSS version 20.0 software using a two-way ANOVA analysis of variance with Students *t*-test. Differences were considered significant at *p* < 0.05 and extremely significant at *p* < 0.01.

## Figures and Tables

**Table 1:** Sequences of the primers used in this study.

Primer	Sequence (5′-3′)
RT-qPCR primers	
*Cg*TLR3-RT-F	TGCCAAAAGCAAATGGTGTGAAT
*Cg*TLR3-RT-R	TTTCCCCCAAAACAAACTTCGTC
*Cg*MyD88-2-RT-F	CAGATAAACCGCTACGACGCCTA
*Cg*MyD88-2-RT-R	ATTTCCGATTCCTTTTGGTGGTC
*Cg*Rel1-RT-F	TCCGCACACCACCTTACAA
*Cg*Rel1-RT-R	CGCCTTTATCTTCAGCCTCT
*Cg*IL17-1-RT-F	GCGAACGCCACAGTGTCAAA
*Cg*IL17-1-RT-R	GACGCTACGAGGAAATACGGAC
*Cg*EF-RT-F	AGTCACCAAGGCTGCACAGAAAG
*Cg*EF-RT-R	TCCGACGTATTTCTTTGCGATGT
ChIP-qPCR primers	
*Cg*TLR3-Pro-F1	CAACATGAATCTCAGCAGACG
*Cg*TLR3-Pro-R1	TTCTTCCCAAACTGCCACA
*Cg*TLR3-Pro-F2	AAGAAGGGGGAGGAGTGCT
*Cg*TLR3-Pro-R2	ATGTGTCTTTAAAAGCCGGTG

## Data Availability

Data are contained within the article.

## Abbreviations

*Cg*IL17-1: *Crassostrea gigas* interleukin 17-1; *Cg*MyD88-2, *Crassostrea gigas* myeloid differentiation factor 88-2; *Cg*Rel1, *Crassostrea gigas* Rel1; *Cg*TLR3, *Crassostrea gigas* Toll-like receptor 3; ChIP-qPCR, chromatin immunoprecipitation followed by qPCR; DMSO, dimethyl sulfoxide; H3K4me3, histone H3 lysine 4 trimethylation; IL, interleukin; MEF, monomethyl fumarate; MTA, 5′-methylthioadenosine; MyD88, myeloid differentiation factor 88; NF-κB, nuclear factor nuclear factor kappa-B; PAMPs, pathogen-associated molecular patterns; PBS, phosphate-buffered saline; PRRs, pattern recognition receptors; TLR, Toll-like receptor; TNF, tumor necrosis factor; RT-qPCR, reverse transcription-quantitative PCR.
